# Clinical and genetic characteristics and natural history of Finnish families with familial exudative vitreoretinopathy due to pathogenic *FZD4* variants

**DOI:** 10.1111/aos.16701

**Published:** 2024-05-05

**Authors:** Laura Lähteenoja, Tapani Palosaari, Timo Tiirikka, Maria Haanpää, Jukka Moilanen, Aura Falck, Elisa Rahikkala

**Affiliations:** ^1^ Research Unit of Clinical Medicine and Medical Research Center Oulu Oulu University Hospital and University of Oulu Oulu Finland; ^2^ Department of Clinical Genetics Oulu University Hospital Oulu Finland; ^3^ Department of Ophthalmology Oulu University Hospital Oulu Finland; ^4^ Department of Clinical Genetics Turku University Hospital Turku Finland

**Keywords:** familial exudative vitreoretinopathy, FEVR, *FZD4*, myopia, natural history, phenotypic variability

## Abstract

**Purpose:**

To report clinical and genetic characteristics of familial exudative vitreoretinopathy (FEVR) in the Finnish population.

**Methods:**

Detailed clinical and genetic data of 35 individuals with heterozygous pathogenic variants in *FZD4* were gathered and analysed.

**Results:**

Thirty‐two individuals with *FZD4* c.313A>G variant and three individuals with *FZD4* c.40_49del were included in the study. The clinical phenotype was variable even among family members with the same *FZD4* variant. Only 34% (*N* = 12/35) of variant‐positive individuals had been clinically diagnosed with FEVR. The median age of the onset of symptoms was 2.3 years, ranging between 0 to 25 years. Median visual acuity was 0.1 logMAR (0.8 Snellen decimal), ranging between light perception and −0.1 logMAR (1.25 Snellen decimal). Most (*N* = 33/35, 94%) were classified as not visually impaired. Despite unilateral visual loss present in some, they did not meet the criteria of visual impairment according to the WHO classification. Two study patients (*N* = 2/35, 6%) had severe visual impairment. The most common FEVR stage in study patient's eyes (*N* = 28/70 eyes, 40%) was FEVR stage 1, that is, avascular periphery or abnormal vascularisation. Most of *FZD4*‐variant‐positive study patient's eyes (*N* = 31/50 eyes, 62%) were myopic. Two individuals presented with persistent hyperplastic primary vitreous expanding the phenotypic spectrum of FEVR. Shared haplotypes extending approximately 0.9 Mb around the recurrent *FZD4* c.313A>G variant were identified.

**Conclusion:**

Most study patients were unaffected or had mild clinical manifestations by FEVR. Myopia seemed to be overly common in *FZD4*‐variant‐positive individuals.

## 1 INTRODUCTION

Familial exudative vitreoretinopathy (FEVR) is an inherited retinal disorder first described in 1969 by Criswick and Schepens (1969). Individuals with FEVR suffer from a maldevelopment of the retinal vasculature, presenting as a failure of peripheral retinal vascularisation. Peripheral avascularity causes retinal ischaemia, which can lead to neovascularisation, exudation, vitreous haemorrhage, dragging of the retina with retinal folds and tractional retinal detachment (Robitaille et al., 2011; Schatz & Khan, 2017). To date, the following pathogenic variants in 14 different genes have been confirmed or suggested to cause FEVR: *FZD4*, *NDP*, *LRP5*, *TSPAN12*, *ZNF408*, *KIF11*, *CTNNB1*, *JAG1*, *RCBTB1*, *ATOH7*, *DOCK6*, *ARHGAP31*, *CTNNA1*, and *SNX31* (Chen et al., 1993; Collin et al., 2013; Kondo et al., 2016; Poulter et al., 2010; Robitaille et al., 2002, 2014; Sun et al., 2019; Tao et al., 2021; Toomes et al., 2004; Wu et al., 2016; Xu et al., 2023; Zhang et al., 2020; Zhu et al., 2021).

Six of the known FEVR‐related genes, *FZD4*, *TSPAN12*, *NDP*, *LRP5*, *CTNNB1*, and *CTNNA1*, code for proteins involved in the Norrin/Wnt signalling pathway. The seven other FEVR‐related genes code for proteins in other signalling pathways associated with proper retinal function. The Norrin/Wnt signalling pathway is involved in many physiological processes of the cell, such as cell survival, migration, and proliferation. It also regulates eye organogenesis and angiogenesis, playing a pivotal role in the vascular development of the eye. *FZD4* codes for frizzled‐4 acting as a receptor for Norrin (Norrie disease protein, NDP) (Nikopoulos et al., 2010; Xu et al., 2004). NDP binds to frizzled‐4 and its coreceptor low‐density lipoprotein receptor‐related protein (LRP5), forming a complex that together with the auxiliary component tetraspanin‐12 (TSPAN12), initiates downstream beta‐catenin signalling, which is one of the major factors affecting retinal angiogenesis (Han et al., 2020).

In this study, we report on eight Finnish families with 35 affected family members with dominantly inherited pathogenic heterozygous *FZD4* variants.

## 2 MATERIALS AND METHODS

### 2.1 Patient recruitment

**FIGURE 1:**
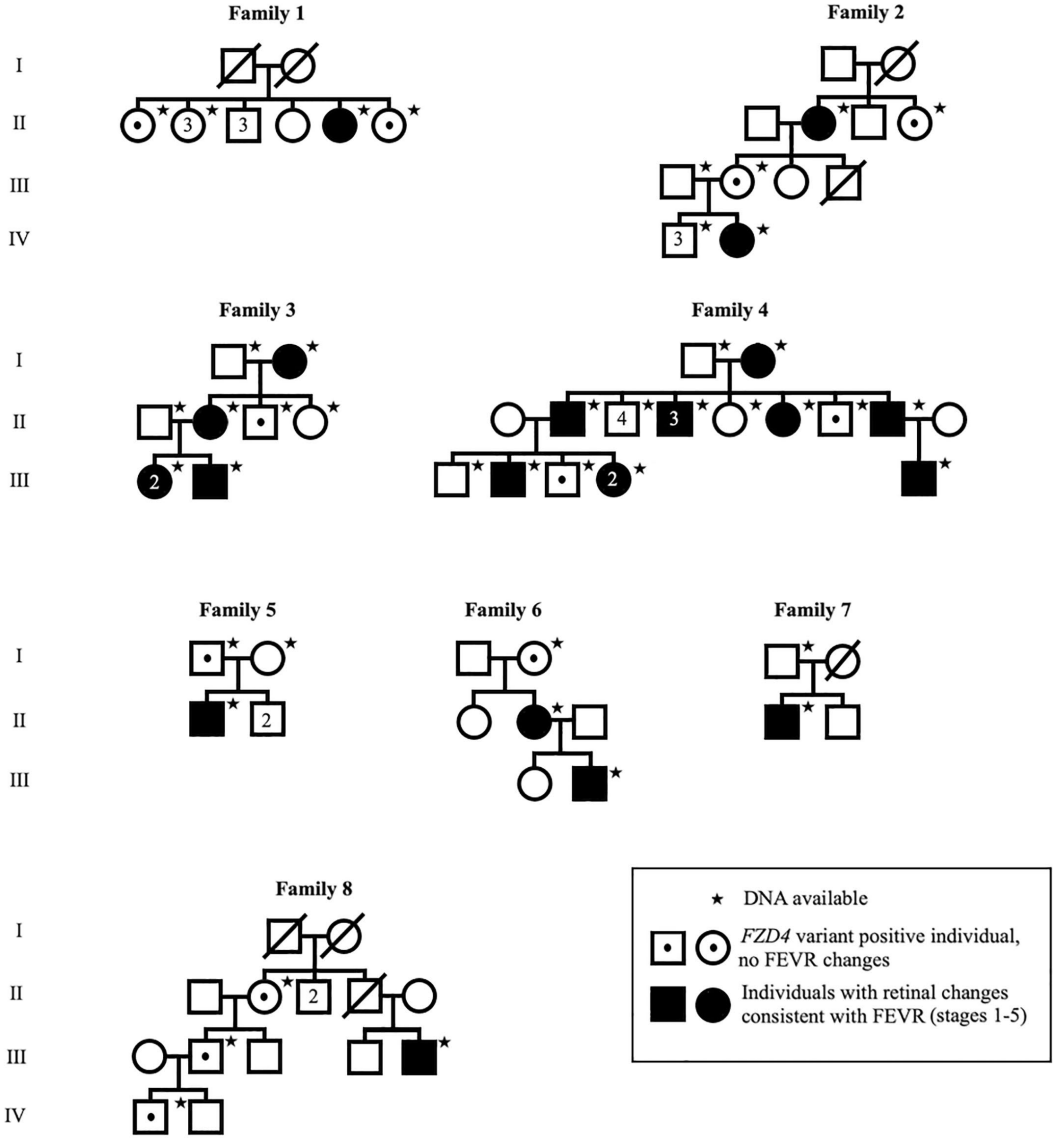
The pedigrees of the eight recruited families. In Families 1–7, the pathogenic variant was *FZD4* c.313A>G, p.(Met105Val), and in Family 8, the pathogenic variant was *FZD4* c.40_40del, p.(Pro14SerfsTer44).

All the probands were suspected to have FEVR and were referred to genetic testing for the molecular genetic confirmation of the suspected diagnosis. Once the known pathogenic causative variant in each family was identified, both affected and unaffected first‐degree relatives of variant‐positive individuals were offered genetic testing of the known familial pathogenic variant. Individuals and their family members with pathogenic or likely pathogenic *FZD4* variants (Figure 1) in the clinical testing were recruited from the departments of clinical genetics in Oulu University Hospital (Families 1–7) and Turku University Hospital (Family 8). Written informed consent was obtained from the participating individuals or from their guardians. This study was conducted in accordance with the Declaration of Helsinki and approved by the ethical review committee of Oulu University Hospital (EETTMK: 45/2015, amendment 2020).

### 2.2 Clinical examination

Clinical data as well as family history were gathered by retrospective review of electronic medical records. The World Health Organization (WHO) classification was used to assess the level of visual impairment as follows: mild visual impairment as visual acuity of the better eye worse than 6/12 to equal to or better than 6/18, moderate visual impairment as worse than 6/18 to equal to or better than 6/60, severe visual impairment as worse than 6/60 to equal to or better than 3/60 and blindness as worse than 3/60 (Burton et al., 2021). The results of standard ophthalmological examination of peripheral retina were available from all 35 study patients. Wide‐field imaging was available for review from 22 study patients (*N* = 22/35, 63%), and of these fluorescein angiographies (FAG) had been performed in 12 study patients (*N* = 12/35). Study patients with genetically confirmed FEVR were classified into five stages by the severity of the findings in the retina (Table 1) (Kashani et al., 2014). Myopia is defined as a refractive error of −0.5 diopter (D) or more, and high myopia as a refractive error greater than −6.0 D.

**TABLE 1:** FEVR classification (Modified from Kashani et al., 2014).

Stage	Description
1	Avascular periphery or abnormal vascularisation
2	Retinal neovascularisation
2A	Without exudate
2B	With exudate
3	Extramacular retinal detachment
3A	Without exudate
3B	With exudate
4	Macula‐involving retinal detachment
4A	Without exudate
4B	With exudate
5	Total retinal detachment

We present the visual acuity both as LogMAR and Snellen decimal values. Commonly used phrases “counting fingers” (CF), “hand movement” (HM) and “light perception” (LP) were converted to numerical logMAR values as follows: CF = 1.9 logMAR, HM = 2.3 logMAR and LP = 2.7 logMAR (Moussa et al., 2021).

Visual acuities could not be determined in four variant‐positive study patients, in three cases due to early age of under 2 years, but there was no clinical suspicion of any problems in visual development. One variant‐positive study patient had age‐related macular degeneration and was excluded from the analysis of FEVR affecting visual acuity. One study patient had an ocular prosthesis from an early age in his left eye because of FEVR and a visual acuity of 0.0 logMAR (1.0 Snellen decimal) in his right eye.

### 2.3 Genetic testing and data analysis

Peripheral blood samples were collected from available and consenting individuals, and genomic DNA was extracted using an automated QIAsymphony device and the Qiagen Qiasymphony DSP DNA Midi Kit (Qiagen, Hilden, Germany). Next‐generation sequencing based gene panels of probands in Families 1, 2, 5, 7, and 8 were performed as part of clinical diagnostics (Blueprint Genetics, Espoo, Finland; Leeds genetics laboratory, Leeds, UK; Prevention genetics laboratory, Marshfield, WI, USA). Sanger sequencing of *FZD4* of probands in Families 3, 4, and 6 was performed to identify the causative pathogenic variant in the family either as part of a research project (Family 4, [Robitaille et al., 2011]) or as part of clinical diagnostics (Families 3 and 6, Genedx Laboratory, Gaithersburg, MD, USA). When the pathogenic variant in the family was already known, targeted Sanger sequencing of the variant was performed. Four unrelated affected individuals from Families 1–4 were exome sequenced, and potentially causative modifying variants and haplotypes were analysed.

Haplotype analysis was performed using the whole exome sequencing (WES) data from four unrelated Finnish study patients from Families 1–4 who carried the pathogenic *FZD4* c.313A>G variant. Single nucleotide variants with a minor allele frequency (MAF) of maximum 0.1 were selected and analysed in a region approximately 2 Mb around the *FZD4* gene (GRCh38 g.11: 85736568–87326523).

Potentially causative modifying variants in the following known FEVR‐related genes *FZD4*, *NDP*, *LRP5*, *TSPAN12*, *ZNF408*, *KIF11*, *CTNNB1*, *JAG1*, *RCBTB1*, *ATOH7*, *DOCK6*, *ARHGAP31*, *CTNNA1*, and *SNX31* were analysed from the WES data.

Variants were classified categorized into five classes (pathogenic, likely pathogenic, VUS, likely benign, and benign) according to the American College of Medical Genetics and Genomics (ACMG) guidelines (Richards et al., 2015).

In the statistical analysis, the student's *t*‐test was used for between group comparisons. Spearman's correlation coefficient (rho) was calculated for testing the correlation between the severity of FEVR and high myopia.

## 3 RESULTS

### 3.1 Clinical characteristics of the patients

The clinical characteristics of the affected cases for each family included in this study are summarized in Table 2, and the pedigrees are shown in Figure 1. Thirty‐five affected individuals from eight different Finnish families were recruited for the study. Eighteen were males, and 17 were females, and their average age was 35 years (range 11 months – 86 years). The severity of FEVR ranged from no changes to the most severe presentations, including a total retinal detachment.

**TABLE 2:** Clinical characteristics of the 35 study patients with pathogenic variants in the *FZD4*.

Age at onset of symptoms (years, 10 patients)		
Median	2.3	
Range	0–25	
First symptom (11 patients)		
Strabismus	8 (73%)	
Abnormal red reflex	3 (27%)	
Myopia	2 (18%)	
Decreased visual acuity	2 (18%)	
Double vision	1 (9%)	
Visual acuity^a^ (61 eyes of 31 patients)^g^		
Median	0.10	
Range	(−0.10) – (2.8)	
WHO classification of visual impairment (35 patients)^b^		
Not visually impaired	33 (94%)	
Mild	0	
Moderate	0	
Severe	2 (6%)	
Blindness	0	
FEVR stages (70 eyes)		
No FEVR‐related retinal abnormalities	22 (31%)	
1 (1A and 1B)	28 (40%)	
2 (2A and 2B)	9 (13%)	
3 (3A and 3B)	5 (7%)	
4 (4A and 4B)	4 (6%)	
5 (5A and 5B)	2 (3%)	
Diagnosis (70 eyes of 35 patients)		
FEVR stage 1–5	48 (69%) eyes	24 (69%) patients
Strabismus^c^	9 (13%) eyes	9 (26%) patients
Amblyopia	7 (10%) eyes	7 (20%) patients
Cataract	9 (13%) eyes	6 (17%) patients
Congenital malformation of vitreous humour	2 (3%) eyes	2 (6%) patients
Anisometropia		2 (6%) patients
Glaucoma	2 (3%) eyes	1 (3%) patients
Other^d^		
Refraction^e^ (50 eyes of 27 patients)		
Hyperopia	7 (14%) eyes	
Myopia	31 (62%) eyes	
Median	−3.1	
Mean	−4.7	
Range	(−0.75) – (−17.5)	
High myopia^f^	10 (20%) eyes	6 (22%) patients
Astigmatism	23 (46%) eyes	
Operations (70 eyes of 35 patients)		
Photocoagulation	15 (21%) eyes	10 (29%) patients
Retinal kryo	7 (10%) eyes	5 (14%) patients
Cataract surgery	7 (10%) eyes	4 (11%) patients
Vitreous surgery for retinal detachment	4 (6%) eyes	2 (6%) patients
YAG‐laser capsulotomy	4 (6%) eyes	3 (9%) patients
Refractive laser	4 (6%) eyes	2 (6%) patients
Strabismus surgery	2 (3%) eyes	2 (6%) patients
Intravitreal VEGF‐inhibitor injections	2 (3%) eyes	1 (3%) patients

^a^ The visual acuity was measured using the logMAR equivalent.

^b^ Burton et al. (2021).

^c^ Constant strabismus.

^d^ Other ophthalmological diagnoses were chronic conjunctivitis (1), chronic uveitis (1), chronic iritis (1), entropium (1) and a benign tumour of the choroidea (1).

^e^ Myopia was defined by a refraction of less than−0.5D, hyperopia was defined by a refraction of more than +0.5D and astigmatism was defined by a refraction of more than +1.0D.

^f^ Pathological myopia was defined by a refraction of less than −6.0D.

^g^ One study patient had an ocular prosthesis because of FEVR and this eye was not taken into account.

**FIGURE 2:**
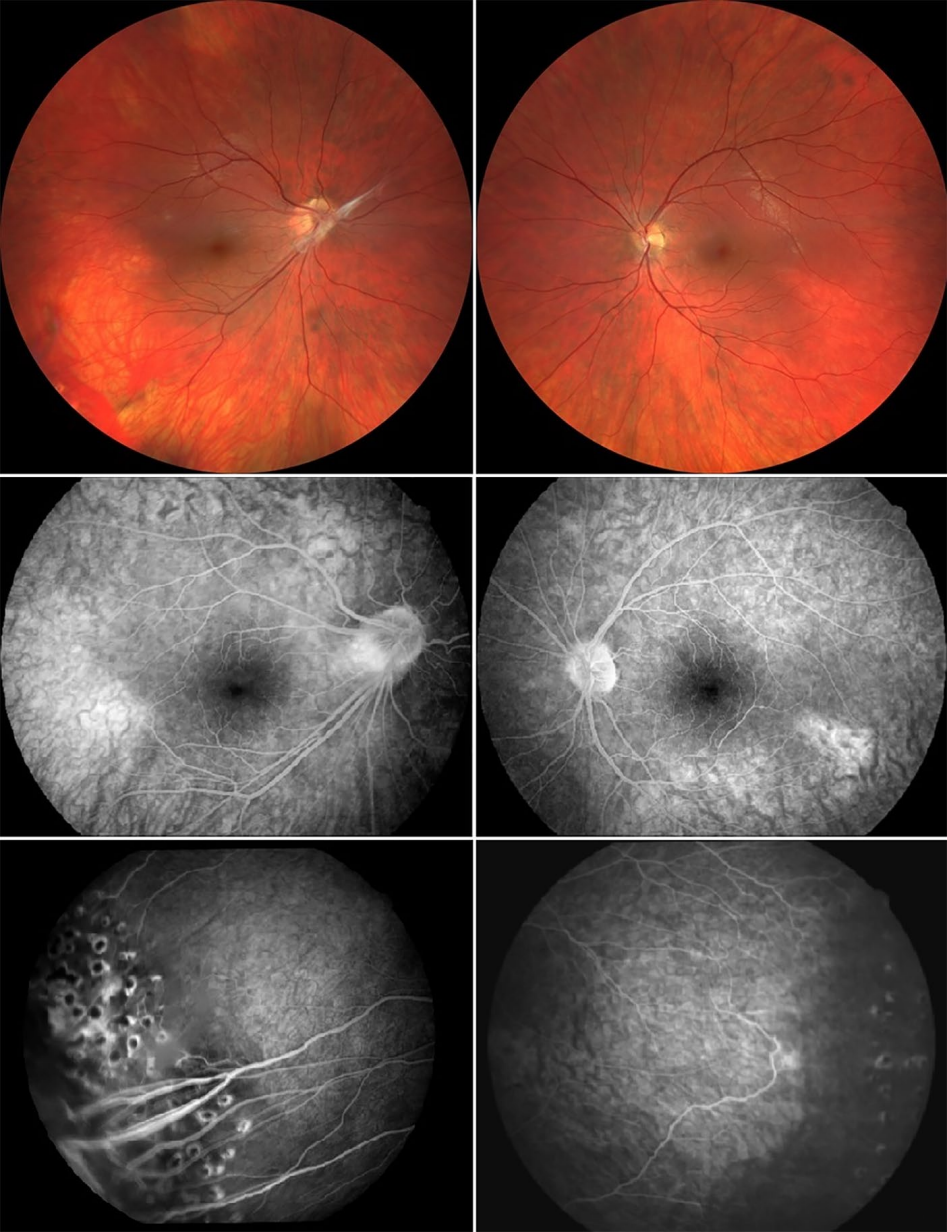
Fundus photographs of the right and the left eye of a female patient with class 3B (right eye) and 2B (left eye) FEVR due to the pathogenic *FZD4* c.313A>G, p.(Met105Val) variant. Photographs above show the fundi at the age of 24 years. FAG images of the central retina at the age of 17 years are shown in the middle and the FAG images at the bottom show retinal periphery treated with laser photocoagulation at the age of 18 years. The visual acuity is 0.6 logMAR (0.25 Snellen decimal) in the right eye and 0.3 logMAR (0.5 Snellen decimal) in the left eye.

Standard ophthalmological examination of peripheral retina had been performed for all participating 35 variant‐positive study patients and 24/35 (69%) of them had retinal changes consistent with FEVR. There were 12 symptomatic study patients with a clinical diagnosis of FEVR, and these subjects had the most severe findings and symptoms among all 35 variant‐positive study patients. There was variability in the clinical presentation between study patients within the families and even within individuals. Variability is demonstrated by Proband 4 (Figure 1, Family 4, II‐11) in Figure 2, who has severe FEVR in the right eye and mild FEVR in the left eye.

The study patients' retinal FEVR changes were divided into FEVR stages 1–5 and no FEVR‐related retinal abnormalities (Kashani et al., 2014). Eleven study patients (*N* = 11/35, 31%) did not have retinal changes related to FEVR. The most common FEVR stage was 1 (*N* = 28/70 eyes, 40%), meaning that the study patients had abnormal retinal vascularisation or peripheral avascularity without neovascularisation, exudates, or other more severe manifestations (Table 2). The more severe FEVR stages 2, 3, 4, and 5 were identified in 13% (*N* = 9/70 eyes), 7% (*N* = 5/70 eyes), 6% (*N* = 4/70 eyes), and 3% (*N* = 2/70 eyes), respectively.

Median visual acuity was 0.10 logMAR (0.8 Snellen decimal), ranging between LP to −0.10 logMAR (1.25 Snellen decimal). The majority of individuals carrying a pathogenic *FZD4* variant (*N* = 33/35, 94%) were classified as not fulfilling the criteria of moderate or severe visual impairment according to the WHO classification (i.e. visual acuity in the better eye was equal to or better than 0.5 logMAR or 0.3 Snellen decimal), and only two study patients (6%, *N* = 2/35) had severe visual impairment. However, among the 33 variant‐positive study patients without moderate or severe visual impairment, eight had unilateral visual loss, that is, visual acuity worse than 0.5 logMAR. Two of the impaired neighbouring eyes had moderate visual impairment, two had severe visual impairment, and four were blind. Thirty‐nine percent of study patients (*N* = 12/31) had a visual acuity better than or equal to 0.1 LogMAR (0.8 Snellen decimal) in their worse eye. Sixty‐nine percent (*N* = 24/35) of variant‐positive individuals had FEVR‐related findings.

The median age of symptom onset was 2.3 years, ranging from 0 to 25 years. The first symptom was most often strabismus (73%), followed by abnormal red reflex (27%), myopia (18%), decreased visual acuity (18%), and double vision (9%). Other common diagnoses were strabismus, amblyopia, cataract, and high myopia. The operations that most often took place were retinal photocoagulation, retinal cryo surgery, and cataract surgery (Table 2).

### 3.2 Natural history of FEVR

To understand the natural history of the disease better, we describe how the study patients developed visual impairment. Two study patients (Figure 1, Proband 5 from Family 5, II‐1 and Proband 8 from Family 8, III‐5) had severe visual impairment.

Proband 5 was diagnosed with FEVR at the age of 9 years. The first ophthalmological examination results available for evaluation were at the age of 23 years, when his visual acuities were at CF level in his right eye and 0.5 LogMAR (0.3 Snellen decimal) in his left eye, and he was diagnosed with cataract in his left eye. By the age of 26 years, he had had multiple retinal laser treatments, multiple vitrectomies, and scleral buckling to both eyes. With each surgery he had peribulbar steroid injections. At 25 years, he had cataract surgery in his left eye and an intraocular lens (IOL) was implanted. At 36 years, he had cataract surgery and IOL implantation in the right eye. Neovascular glaucoma (NVG) was diagnosed at the age of 26 years. NVG treatments included laser iridotomies and surgical iridectomies. At 28 years of age, he was diagnosed with chronic uveitis and received oral methotrexate and prednisolone in addition to local therapy. The most recent ophthalmological examination at the age of 37 years revealed visual acuity 1.2 LogMAR (0.063 Snellen decimal) in the right eye and LP in the left eye.

Proband 8 had multiple vitrectomies and retinal laser treatments in both eyes to treat retinal detachments by the age of 18 years. The study patient also had scleral buckling in his right eye. The first available visual acuity was at 18 years of age, 1.3 LogMAR (0.05 Snellen decimal) in the right eye and HM in the left eye. By this age he had had cataract surgery of the right eye. During the following years, he had vitrectomies and retinal laser treatments in his right eye. He was completely blind in his left eye at the age of 25 years old. The latest measured visual acuity was 1.3 LogMAR (0.05 Snellen decimal) in his right eye. The study patient had YAG‐laser capsulotomy in his right eye at the age of 36 years.

**FIGURE 3:**
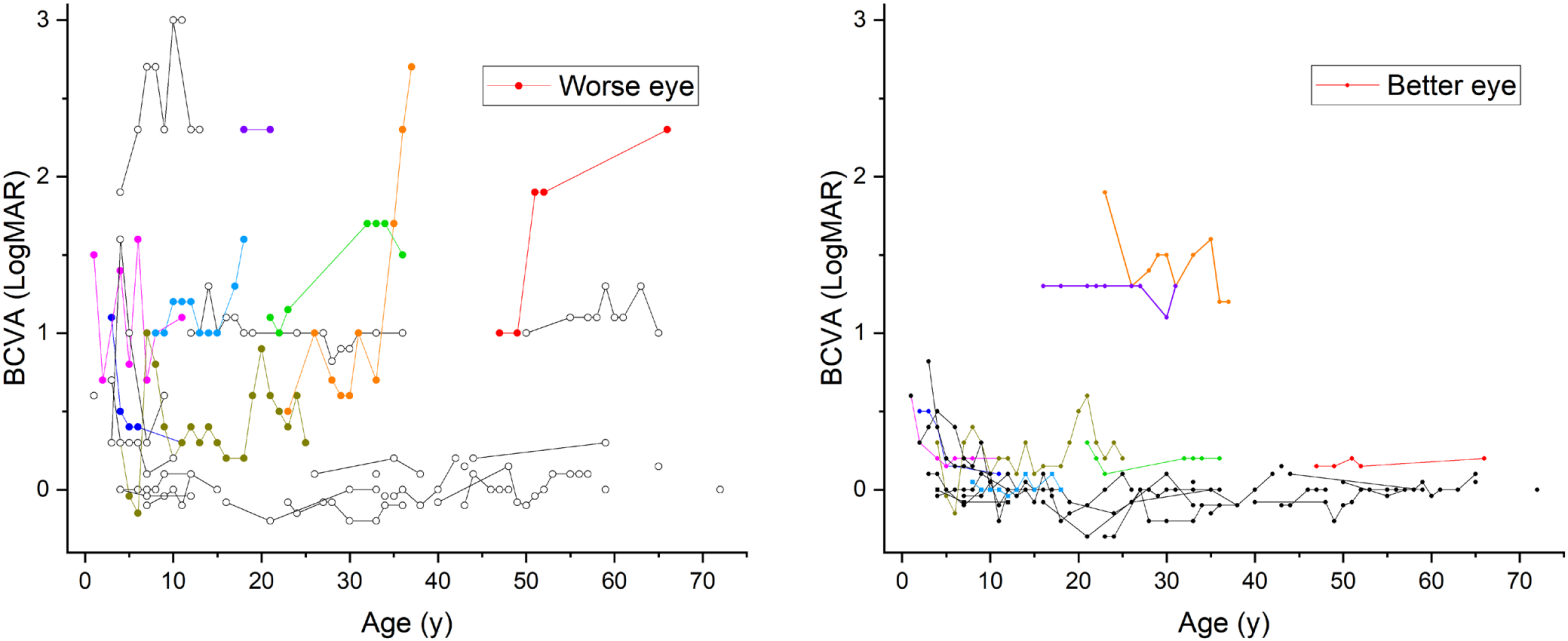
The best corrected visual acuities (BCVA) in the worse eye and better eye at different ages are shown. Probands from Families 1–8 are in colour and *FZD4* variant‐positive family members are in black. In most individuals, the BCVA remained constant in the follow‐up, and only a few individuals experienced significant deterioration of BCVA. In this cohort, only two individuals had bilaterally severe visual impairment. One study patient had an ocular prosthesis because of FEVR, and this eye was not taken into account.

For all 35 *FZD4* variant‐positive study patients the known natural history of the best corrected visual acuity (BCVA) is demonstrated in Figure 3.

**FIGURE 4:**
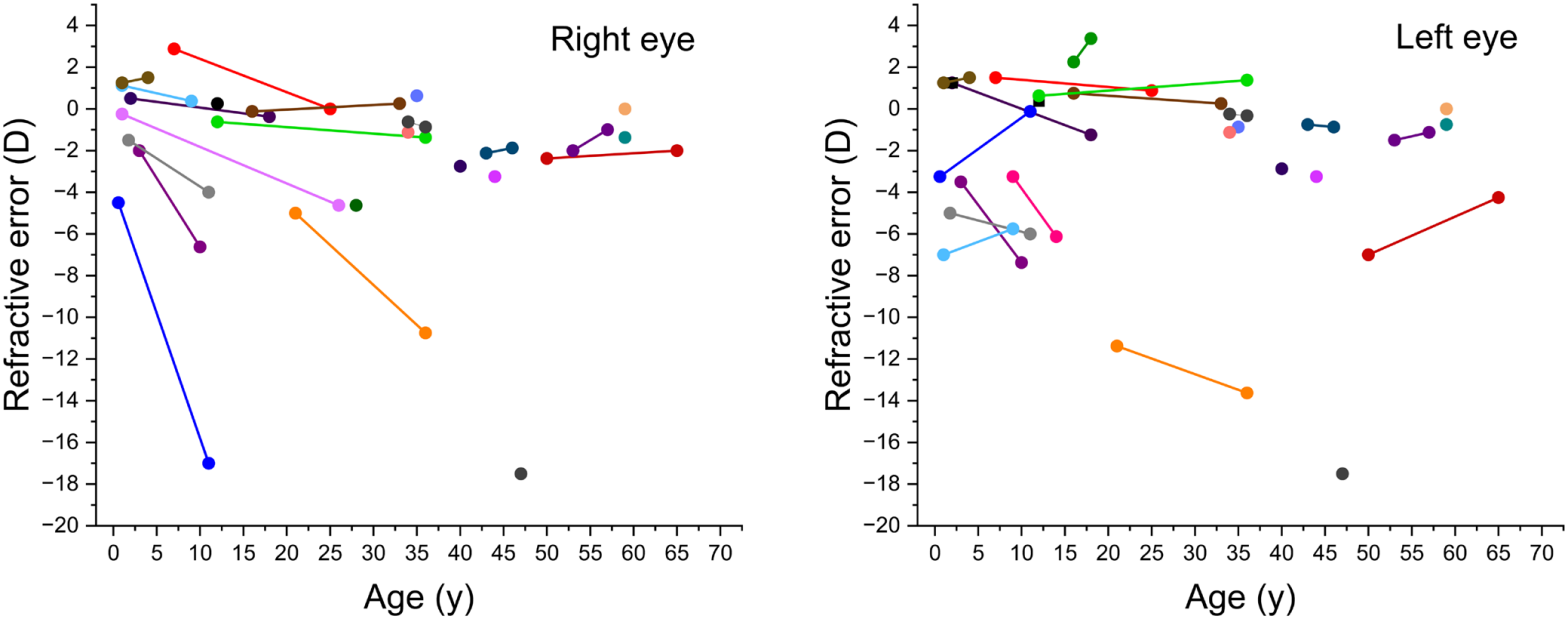
*FZD4*‐variant‐positive individuals' first and last available refractive errors are presented with lines and circles. For some individuals, only one measurement of refractive error was available (circles). Different colours represent different individuals. Some study patients had abnormally high myopia at a relatively young age.

Six study patients (*N* = 6/27, 22%) had pathological myopia in 10 eyes (*N* = 10/50, 20%) defined by a refractive error greater than −6.0 D. We compared the refractions in study patients' eyes classified in FEVR stages 0–1 and 2–4 using student's *t*‐test. No difference was found between the subjects with no or mild manifestations of FEVR and those with more severe FEVR, the mean difference in refractions being −0.092 (95% CI −3.1 to 2.9, *p* > 0.90). Spearman's correlation coefficient showed no correlation between the severity of FEVR and high myopia (rho = −0.066). Study patients' first and last available refractions are shown in Figure 4, which elaborates the natural history of FEVR.

### 3.3 Genetic results

**FIGURE 5:**
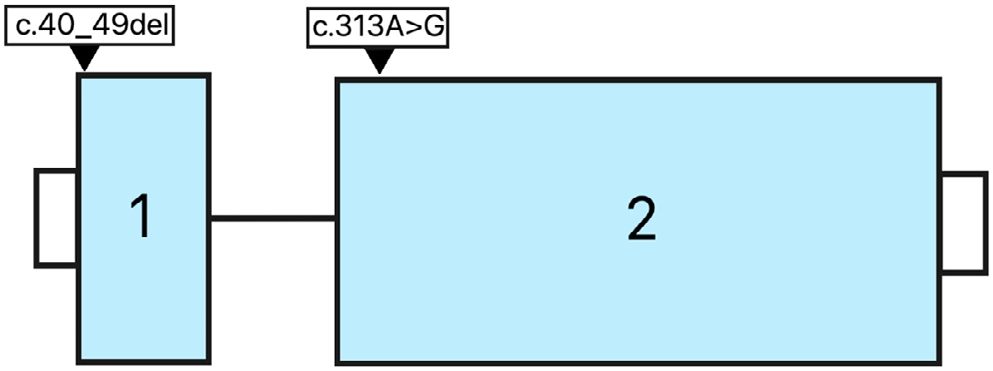
The exons of the *FZD4* gene and locations of the *FZD4* c.40_40del and c.313A>G variants. Exons are to scale, whereas the introns and UTRs are not to scale. The isoform reference is *FZD4*: NM_012193.4, ENST00000531380.2.

Thirty‐two individuals from Families 1–7 were heterozygous for the *FZD4* (NM_012193.4) c.313A>G, p.(Met105Val) variant (hg38: chr11:86952443T>C, rs80358284) (Figure 5), which was classified as pathogenic according to the ACMG guidelines (PP5_supporting PM1_moderate, PM5_moderate, PS3_moderate, PP1_strong) (Richards et al., 2015). The variant is present in gnomAD, including 31 heterozygous individuals, and its MAF is 9.2 times higher in the Finnish population (0.0001252) than in non‐Finnish Europeans (0.00001356) (gnomAD v.4.0.0, accessed on 21st March 2024) (Karczewski et al., 2020).

The individuals in Family 8, from western Finland, who were affected by FEVR, were heterozygous for *FZD4* (NM_012193.4) c.40_49del, p.(Pro14SerfsTer44) variant (hg38: chr11:86955037‐CGCCCCCGGG, rs1555086007), which is classified as pathogenic according to the ACMG guidelines (PVS1_very strong, PP5_supporting, PM2_supporting). It was present in gnomAD in four heterozygous individuals.

In addition to the pathogenic *FZD4* p.(Met105Val) variant, the Proband in Family 2, carried also a paternally inherited *TSPAN12* (NM_012338.4) c.612G>A, p.(?) variant (hg38: chr7:120806549C>T, rs755636404), which is classified as a VUS (PP3_moderate, PM2_supporting). No other possible modifying factors were identified in the next‐generation sequencing based gene panels or exome analysis.

### 3.4 Haplotype results

Haplotype analysis of the recurrent *FZD4* c.313A>G, p.(Met105Val) variant revealed shared haplotypes extending ~ 0.9 Mb around the variant (Table S1).

## 4 DISCUSSION

In this study, we report clinical characteristics of 35 individuals from eight Finnish families with a pathogenic *FZD4* variant. In our family‐based study, where the family members were ascertained by cascade testing of symptomatic and asymptomatic individuals, we aimed to report the phenotype of individuals with a pathogenic variant in *FZD4* without the necessity of clinically diagnosed FEVR. To our knowledge, this study is the first cohort where the inclusion criterion was solely the presence of a pathogenic variant in *FZD4*. In our cohort, most study patients had mild clinical manifestations and either mild or no visual impairment. Forty percent of individuals presented with mild findings, such as abnormal vascularisation or peripheral avascularity without neovascularisation, exudates, or other more severe manifestations 31% of study patients had no FEVR‐related retinal abnormalities. And 53% of study patients were classified as mild FEVR (stage 1 or 2). There was a marked clinical variability in the clinical presentation between different study patients, and some individuals had asymmetry between their eyes (Figures 2 and 3).

In 2022, Wang et al. published a systematic review and meta‐analysis on more than 3200 FEVR patients. Out of these patients, there were 109 *FZD4*‐variant‐positive FEVR patients with clinical staging of the severity of the disease. Only 21.4% of the patients had mild FEVR (stages 1–2), and 76.8% had severe disease (stages 3–5). The meta‐analysis suggested that *FZD4* is causal to the more severe form of FEVR compared with other FEVR‐associated genes (Wang et al., 2022). In our family‐based study, where also asymptomatic family members had been recruited and genetically tested, the majority of the variant‐positive study individuals' eyes (*N* = 37/70 eyes, 53%) had mild FEVR or no manifestation of FEVR (*N* = 22/70 eyes, 31%), demonstrating that the prevalence and severity of the disease depends on the recruitment and testing criteria. Sixty‐nine percent (*N* = 24/35) of variant‐positive individuals had FEVR‐related findings in their eyes. The most common FEVR classification was 1 (*N* = 28/70 eyes, 40%), meaning that even the patients who do have retinal fundus findings related to FEVR most likely had mild ones, such as abnormal retinal vascularisation or peripheral avascularity without neovascularisation, exudates, or other more severe manifestations (Table 2).

Interestingly, we found that myopia was a common finding among *FZD4* variant‐positive individuals. Sixty‐two percent of variant‐positive individuals' eyes were myopic (*N* = 31/50 eyes), while the prevalence globally for myopia is around 30% and in Finland 21%–30% (Holden et al., 2016; Pärssinen, 2012). The median refractive error of *FZD4* variant‐positive individuals was −3.1 D, and some study patients had abnormally high myopia at a relatively young age (Figure 4). This finding is in line with previous studies that found myopia and high myopia from a young age in FEVR patients (Chen et al., 2022; Yang et al., 2002). Our study most likely underestimates the risk for myopia in our study patients as two study patients had had refractive laser surgery in both eyes and three study patients had had cataract surgery in four eyes. These eyes were not taken to account because the refractive errors before surgeries were not available.

In our study, individuals with either the *FZD4* c.313A>G variant or the *FZD4* c.40_49del variant showed marked variability in the ophthalmological manifestations. Approximately, a third of variant‐positive individuals (31%, *N* = 11/35) did not have any retinal changes suggestive of FEVR demonstrating incomplete penetrance associated with *FZD4* variants. However, we cannot exclude the possibility that mild changes may have been missed in standard fundus examination, especially in young children.

The *FZD4* c.313A>G variant is one of the most frequently reported pathogenic variants causing FEVR in different populations (Xiao et al., 2019), and it is also the most prevalent variant in the Finnish population. The *FZD4* c.313A>G variant is enriched in the Finnish population, with an MAF nine times higher in Finns than in non‐Finnish Europeans. Our haplotype analysis of four unrelated individuals showed a shared haplotype extending 0.9 Mb around *FZD4* c.313A>G, suggesting that this variant may be a founder variant in the Finnish population. It is also possible that nucleotide 313 of *FZD4* is a mutational hot spot, explaining its presence in different populations.

*FZD4* c.313A>G has also been reported in seven control individuals in the gnomAD database, demonstrating a clinical heterogeneity of FEVR ranging between small avascular areas at the peripheral retinal only visible by wide‐angle fundus FAG and severe sight‐threatening complications such as retinal fold and detachment, vitreous haemorrhage and macular ectopia. It is possible that epigenetic, modifying genetic and stochastic tissue‐ and timing‐specific factors during the development influence the penetrance and severity of FEVR. A recent study suggests that the *FZD4* exon 1 methylation level may be negatively linked with FEVR severity, suggesting that epigenetic factors also influence the severity of FEVR (Liu et al., 2022).

Both *FZD4* variants reported in this study are expected to cause loss‐of‐function of frizzled‐4. The *FZD4* c.40_49del variant creates a premature stop codon and is expected to result in an absent or truncated protein product, leading to haploinsufficiency. The *FZD4* c.313A>G, p.(Met105Val) variant is located in the conserved N‐terminal extracellular cysteine‐rich domain of frizzled‐4 essential to ligand recognition (Xiao et al., 2019). As *FZD4* residue M105 is one of the key residues comprising a hydrophobic core at the binding interface, it has been speculated that the *FZD4* c.313A>G, p.(Met105Val) variant interrupts the binding of Norrin to frizzled‐4 (Shen et al., 2015). Defective trafficking resulting in haploinsufficiency has been suggested as a cellular mechanism for pathogenic *FZD4* missense variants causing FEVR (Milhem et al., 2014).

In summary, this study demonstrated that *FZD4* c.313A>G is the prevalent pathogenic variant in the Finnish population. The majority (94%, *N* = 33/35) of individuals carrying a pathogenic *FZD4* variant were classified as visually able and did not fulfil the WHO criteria for moderate or more severe visual impairment. However, of these 33 study patients, eight (24%, *N* = 8/33) had visual loss to some extent at least in one of their eyes. Two had moderate visual loss, two had severe visual loss and four were unilaterally blind due to FEVR. Only two study patients (6%, *N* = 2/35) had bilateral severe visual impairment, which can be taken to account when counselling patients on the prognosis of FEVR.

## FUNDING INFORMATION

This study was funded by the Research Council of Finland [grant number 338446], Juhani Ahon Lääketieteen Tutkimussäätiö, Sokeain Ystävät – De Blindas Vänner sr, Suomen Kulttuurirahasto (Ingrid, Toini and Olavi Martelius fund), Pohjois‐Suomen terveydenhuollon tukisäätiö (Terttu foundation), Suomen Lääketieteen säätiö [grant number 4967], Retinary and State Research Funding for the Oulu University Hospital.

## CONFLICT OF INTEREST STATEMENT

None of the authors declares any conflicts of interest.

## Supporting information


Data S1:
